# The Emerging Role of Poly (ADP-Ribose) Polymerase Inhibitors as Effective Therapeutic Agents in Renal Cell Carcinoma

**DOI:** 10.3389/fonc.2021.681441

**Published:** 2021-07-09

**Authors:** Jerred P. Pletcher, Sayani Bhattacharjee, Jonathan P. Doan, Rebecca Wynn, Puneet Sindhwani, Nagalakshmi Nadiminty, Firas G. Petros

**Affiliations:** ^1^ College of Medicine and Life Sciences, The University of Toledo, Toledo, OH, United States; ^2^ Graduate Program in Cancer Biology, The University of Toledo, Toledo, OH, United States; ^3^ Department of Urology, The University of Toledo, Toledo, OH, United States; ^4^ Department of Cancer Biology, The University of Toledo, Toledo, OH, United States

**Keywords:** renal cell carcinoma, kidney cancer, therapy, PARP inhibition, DNA damage repair

## Abstract

Renal cell carcinoma (RCC) is the sixth most common cancer in the US. However, no significant changes in management have occurred since the tyrosine kinase era until the recent breakthrough with checkpoint inhibitors. Therefore, the need for more therapeutic options is paramount. Our objective was to determine whether PARP inhibition represents a novel therapeutic option for RCC. We used publicly available COSMIC, GDC Data Portal, and cBioPortal databases to explore mutations in DNA repair genes in RCC tissues from the TCGA cohort. We treated a human normal renal epithelial cell line RPTEC/TERT1 and two human renal cancer cell lines ACHN and CAKI-2 with PARPi niraparib, olaparib, rucaparib, veliparib, and talazoparib. Cell survival, cell proliferation, clonogenic ability, and apoptosis were assessed. RCC xenografts in SCID mice were treated with PARPi to evaluate their efficacy *in vivo*. Data mining revealed that ~27-32% of RCC tissues contain mutations in homologous recombination genes. Niraparib and talazoparib were the most effective at reducing cell survival, proliferation, and clonogenic ability *in vitro*. Niraparib, talazoparib, and rucaparib were the most effective in reducing RCC xenograft growth *in vivo*. Agents such as PARPi that exploit mutations in DNA damage repair genes may be effective therapeutic options for RCC.

## Introduction

Renal cell carcinoma (RCC) is the ninth most prevalent cancer world-wide and in the US (1). Clear-cell RCC (ccRCC) represents approximately 70% of RCC histology, while papillary RCC (pRCC), and chromophobe RCC (chRCC) account for the majority of non-ccRCC subtypes (2). Most patients present with localized disease, and definitive local treatment remains the gold standard for patients with no distant metastases (3, 4), while systemic drug therapy is an established practice for metastatic disease (5–7). The US Food and Drug Administration (FDA) has approved several systemic therapies including anti-angiogenic agents, mammalian target of Rapamycin (mTOR) inhibitors, and immunotherapeutic agents for the treatment of RCC (8). While the overall survival of patients with metastatic RCC has improved with these systemic therapies, disease progression and mortality are inevitable in most patients with advanced RCC. Hence, novel therapeutic options are urgently needed.

Genomic DNA damage results from several hits such as free radicals generated during cellular metabolism or environmental carcinogens. Damaged DNA is repaired by the Poly (ADP) Ribose Polymerase (PARP) family proteins. PARP binding to sites of single-strand breaks (SSB) results in the repair of the SSB. In PARP-deficient cells, SSB can be converted to double-strand breaks (DSB) and are repaired by DSB repair mechanisms such as homologous recombination (HR) and non-homologous end rejoining (NHEJ). BRCA1 and BRCA2 are an integral part of the HR pathway. NHEJ is comprised of several sub-types based on the polymerases, nucleases, and ligase complexes involved including alternative NHEJ and microhomology-mediated end-joining (9, 10). Cells deficient in BRCA proteins rely on the highly error prone NHEJ for DNA repair, which often leads to instability and transformation. Cells deficient in both BRCA and PARP are subject to “synthetic lethality”. Hence, it has been recognized that tumor cells deficient in BRCA proteins may be selectively targeted by PARP inhibitors (PARPi). Due to the low frequency of germline BRCA mutations or loss in urological malignancies, PARPi were thought to be of low utility in urologic cancers. However, recent studies suggest that the efficacy of PARPi does not depend only on BRCA loss, but deficiency or mutations in other components of the HR pathway may also mimic BRCA loss and synthetic lethality, known as “BRCAness” (11).

RCC occurs as hereditary as well as sporadic cancers. Hereditary ccRCC is associated with alterations in the Von Hippel-Lindau (VHL) gene, leading to the increased expression of the angiogenic factor VEGF, whereas hereditary pRCC is well known for its association with the c-MET oncogene (12–14). ccRCC is also associated with the chromosome 3p translocation and gain of 5q. PBRM1, BAP1, and SETD2, genes encoding histone and chromatin regulators, are present on chromosome 3p and are mutated at frequencies of 38%, 11%, and 13.2%, respectively in ccRCC (15) and at frequencies of 4.5%, 6.4%, and 5.6% in pRCC. chRCC is associated with the Birt-Hogg-Dube syndrome and carries a significantly lower mutation rate compared with ccRCC and pRCC (15). Mutations in genes such as p53, PTEN, and mitochondrial DNA mutations are associated with the majority of chRCC cases (15, 16). Leiomyomatosis syndrome, tuberous sclerosis complex, and succinate dehydrogenase gene mutations also predispose to rare forms of early-onset hereditary RCC (14, 17). In addition, RCC is characterized by somatic loss of function mutations in DNA damage repair genes such as TTN, MUC4, MUC16, DST, KMT2C, KMT2D, and ARID1A (15). Notwithstanding the recognition that RCC harbors several defects in DNA damage repair (18, 19), studies on the susceptibility of RCC to PARPi are scarce.

The current study aims to provide evidence for the role of DNA damage repair defects in the success of PARPi in RCC. Understanding the mechanisms and the association of HR repair defects with sensitivity to PARPi can signal a breakthrough in RCC therapy. We demonstrate that PARPi can be therapeutics of choice in cancers such as RCC that may not possess mutations in the classical HR gene BRCA.

## Materials and Methods

### Mutational Analysis

Publicly available TCGA datasets containing mutational data for genes in the Homologous Recombination (HR) pathway were queried using COSMIC, The Cancer Genome Atlas, and cBioPortal (https://cancer.sanger.ac.uk/cosmic, https://cancergenome.nih.gov/, and http://www.cbioportal.org/). We sought to determine the prevalent rate of mutations (including frame-shift, missense, nonsense, and deletion) in DNA damage repair pathway genes. COSMIC, the Catalogue of Somatic Mutations in Cancer, is the world’s largest and most comprehensive resource for exploring the impact of somatic mutations in human cancer. The GDC Data Portal, developed by the National Cancer Institute, provides a platform for efficiently querying and downloading high quality and complete genomic data. The cBioPortal for Cancer Genomics provides visualization, analysis and download of large-scale cancer genomics data sets. The list of indirect and direct DNA damage repair genes for which we sought to determine mutational status is in **Table 1**. We used a comprehensive list of genes shown to be involved directly or indirectly in DNA damage repair (20).

**Table 1:** List of all 193 DNA repair genes analyzed.

Direct DNA repair genes				
*ATM 4*	*RPA4 3, 4*	RFC2 1, 2, 3	PCNA 1, 2, 3	*MLH1 2*
*ATR 5*	*BRCA1 4*	RFC3 1, 2, 3	*PARP1 1*	*MSH6 2*
*RPA1 3, 4*	*BRCA2 4, 5*	RFC4 1, 2, 3	*ERCC1 3, 5*	*MSH2 2*
*RPA2 3, 4*	*RAD51 4*	RFC5 1, 2, 3	*MSH3 2*	*MLH3 2*
*RPA3 3, 4*	RFC1 1, 2, 3	*XRCC1 1*	*PMS2 2*	*EXO1 2*
*NBN 4*	*ERCC3 3*	POLD1 1, 2, 3	*APEX2 1*	*ERCC8 3*
*RAD50 4*	*XPA 3*	*MRE11A 4*	*PNKP 1*	*UVSSA 3*
*CHEK2 4*	*RAD23B 3*	*RAD51D 4*	*APLF 1*	*XAB2 3*
*FANCI 5*	*PALB2 4, 5*	*RAD52 4*	*PARP3 1*	*MMS19 3*
*FANCD2 5*	*RAD51C 4, 5*	*RAD51B 4*	*ALKBH2 7*	*DMC1 4*
*FANCA 5*	*XRCC6 6*	*PMS1 2*	*ALKBH3 7*	*XRCC2 4*
*FANCC 5*	*XRCC5 6*	*RAD23A 3*	*MSH4 2*	*XRCC3 4*
*FANCE 5*	*PRKDC 6*	*LIG3 1, 3*	*MSH5 2*	*RAD54L 4*
*FANCL 5*	*XRCC4 6*	*MGMT 7*	*PMS2P3 2*	*RAD54B 4*
*FANCG 5*	*Lig4 6*	*OGG1 1*	*CETN2 3*	*SHFM1 4*
*FANCM 5*	*FANCB 5*	*UNG 1*	*DDB1 3*	*RBBP8 4*
*ERCC4 3*	*FANCF 5*	*SMUG1 1*	*DDB2 3*	*SLX1A 4*
*ERCC2 3*	*FAAP24 5*	*MBD4 1*	*GTF2H1 3*	*SLX1B 4*
*ERCC5 3*	*CHEK1 5*	*TDG 1*	*GTF2H3 3*	*GEN1 4*
*PARP2 1*	*BRIP1 5*	*MUTYH 1*	*GTF2H4 3*	*FAAP20 5*
*APEX1 1*	*SLX4 5*	*NTHL1 1*	*GTF2H5 3*	*DCLRE1C 6*
*FEN1 1*	*FAN1 5*	*MPG 1*	*CDK7 3*	*NHEJ1 6*
*XPC 3*	*MUS81 4, 5*	*NEIL1 1*	*CCNH 3*	
*ERCC6 3*	*EME1 4, 5*	*NEIL2 1*	*MNAT1 3*	
*GTF2H2 3*	*POLE 1, 3*	*NEIL3 1*	*LIG1 1, 3*	
Indirectly associated genomic stability maintenance genes				
*PAXIP1*	*AURKB*	*POLI*	*SHPRH*	*DCLRE1A*
*BLM*	*POLB*	*POLK*	*HLTF*	*DCLRE1B*
*MLL3*	*POLH*	*POLL*	*RNF168*	*PRPF19*
*CRIP1*	*POLQ*	*POLM*	*SPRTN*	*RECQL*
*CDK12*	*TDP1*	*POLN*	*RNF8*	*RECQL5*
*BAP1*	*TDP2*	*TREX1*	*RNF4*	*HELQ*
*BARD1*	*NUDT1*	*TREX2*	*UBE2V2*	*RDM1*
*WRN*	*DUT*	*APTX*	*UBE2N*	*NABP2*
*BUB1*	*RRM2B*	*SPO11*	*H2AFX*	*ATRIP*
*CENPE*	*POLG*	*ENDOV*	*CHAF1A*	*MDC1*
*ZW10*	*REV3L*	*UBE2A*	*SETMAR*	*RAD1*
*TTK*	*MAD2L2*	*UBE2B*	*RECQL4*	*RAD9A*
*KNTC1*	*REV1*	*RAD18*	*MPLKIP*	*HUS1*
*RAD17*	*TP53BP1*	*CLK2*		
*TP53*	*TOPBP1*	*PER1*		

Classified as direct DNA repair genes or indirect regulators of genomic stability. Direct DNA repair genes further classified by involved repair pathway or pathways (1 = BER, 2 = MMR, 3 = NER, 4 = HR, 5 = FA, 6 = NHEJ, 7 = DR). Adapted from (20).

### Cell Lines and Reagents

Human RCC cell lines Caki-2 (representing ccRCC), ACHN (representing pRCC) and the normal human renal epithelial cell line RPTEC/TERT1 were obtained from the American Type Culture Collection (ATCC, Manassas, VA), and cultured in McCoy’s 5a modified medium with 10% complete FBS; Eagle’s Minimum Essential Medium with 15% FBS; and DMEM: F12 medium supplemented with hTERT RPTEC Growth kit as recommended by ATCC, respectively. All experiments with cell lines were performed either within 6 months of receipt from ATCC or resuscitation after cryopreservation. ATCC uses Short Tandem Repeat (STR) profiling for testing and authentication of cell lines. Antibodies against Tubulin were from Thermo Fisher Scientific. Antibodies against cleaved caspase-3, cleaved caspase-7, cleaved caspase 9, cleaved PARP, whole caspase 3, whole caspase 7, whole caspase 9, and whole PARP were from Cell Signaling Technologies. PARP inhibitors (niraparib, olaparib, rucaparib, talazoparib, and veliparib) were obtained from MedChem Express. All other reagents were of analytical grade and obtained from local suppliers.

### Cell Growth Assays

Cells were treated with PARPi as indicated in the figures. Viable cell numbers were determined using a Coulter cell counter (Beckman Coulter).

### Cell Proliferation Assays

Cells were treated with PARPi for 72 h. Cell proliferation was assessed using the CellTiter 96 Aqueous One Solution Cell Proliferation assay (Promega) according to the manufacturer’s instructions.

### Clonogenic Assays

Anchorage-dependent clonogenic ability assays were performed as described previously (21). Briefly, cells were treated with varying concentrations of PARPi for 72 h. Cells were trypsinized and replated at low densities (400 cells/well) in 6-well plates in triplicate. The plates were incubated at 37°C undisturbed for 10-14 days. At the end of the experiment, colonies were stained with 0.5% Crystal Violet in buffered formalin and the numbers of colonies were counted.

### Western Blot Analysis

Cells were lysed in high salt buffer containing 50 mM Hepes pH 7.9, 250 mM NaCl, 1 mM EDTA, 1% NP-40, 1 mM PMSF, 1 mM Na Vanadate, 1 mM NaF and protease inhibitor cocktail (Roche). Total protein was estimated using the Coomassie Protein Assay Reagent (Pierce). Equal amounts of protein were loaded on 10% SDS–PAGE and transferred to nitrocellulose membranes. The membranes were blocked with 5% nonfat milk in PBST (1x PBS+0.1% Tween-20) and probed with the indicated primary antibodies in 1% BSA. The signal was detected by ECL (Millipore) after incubation with the appropriate HRP-conjugated secondary antibodies.

### Mouse Xenograft Assays

RCC cells (2x10^6^) were injected along with 1:1 matrigel sub-cutaneously into the flanks of male SCID mice and tumor growth monitored. When the tumor volumes reached approximately 0.1 cm^3^, mice were randomly divided into 10 groups (n=5) and treated with: vehicle (0.5% Methocel A4M), or the indicated doses of PARPi: 10 or 20 mg/kg niraparib; 25 or 50 mg/kg olaparib; 50 or 100 mg/kg rucaparib; 0.5 or 1 mg/kg talazoparib; or 100 mg/kg veliparib. Treatments were performed for ~5 weeks and tumor growth was monitored using digital calipers. At the end of the experiment, tumor tissues were harvested, and the levels of the proliferation marker Ki-67, and cleaved caspases 3, 7, and 9 were analyzed using immunohistochemical staining. The study was conducted in accordance with the animal care and use guidelines of the University of Toledo Institutional Animal Care and Use Committee.

### Immunohistochemistry

Tumors were fixed using formalin and paraffin embedded tissue blocks were dewaxed, rehydrated, and endogenous peroxidase activity blocked. Antigen retrieval was performed in sodium citrate buffer (0.01 mol/L, pH 6.0) in a microwave oven at 1,000 W for 3 min and then at 100 W for 20 min. Nonspecific antibody binding was blocked by incubating with 10% fetal bovine serum in PBS for 30 min at room temperature. Slides were then incubated with anti-Ki-67 (NeoMarker) or indicated antibodies at 4°C overnight. Slides were washed and incubated with biotin-conjugated secondary antibodies for 30 min, followed by incubation with avidin DH-biotinylated horseradish peroxidase complex for 30 min (Vectastain ABC Elite Kit, Vector Laboratories). The sections were developed with the diaminobenzidine substrate kit (Vector Laboratories) and counterstained with hematoxylin.

### Statistical Analyses

Data are shown as means ± SD. Multiple group comparison was performed by one-way ANOVA. P ≤ 0.05 was considered statistically significant.

## Results

### RCC Tissues Harbor Mutations in Several Direct and Indirect DNA Damage Repair Genes

We analyzed genomic data from the TCGA datasets using COSMIC, GDC Data portal, and cBioPortal to assess the rate of mutations in several direct and indirect DNA damage repair genes in RCC clinical tissues. The genes are listed in **Table 1**. The results showed that in addition to genes such as VHL, BAP1, PBRM1, CDKN2A, SET2D, p53, mTOR, and PTEN, RCC tissues harbor mutations in several other DNA damage repair genes (**Table 2**). These results confirmed that RCC tissues harbor relatively high rates of mutations in DNA damage repair genes as shown by previous studies (18, 19). Based on these findings, we aimed to test whether PARP inhibition would be a viable therapeutic strategy for RCC using ccRCC and pRCC cell lines.

**Table 2:** COSMIC, GDC Data Portal, and cBioPortal were used to explore the rate of mutations in direct and indirect DNA repair genes in RCC tissues from the TCGA cohort.

Direct and Indirect DNA repair Genes	COSMIC	GDC Data Portal	cBioPortal
**VHL**	**39.97%**	**27.15%**	**21.3%**
**PBRM1**	**23.50%**	**23.21%**	**20.5%**
**TTN**	**9.03%**	**22.48%**	**0**
**MUC4**	**2.24%**	**12.85%**	**0**
**SETD2**	**9.14%**	**9.49%**	**8.4%**
**MUC16**	**5.43%**	**9.20%**	**0.1%**
**BAP1**	**8.88%**	**7.45%**	**6.4%**
**DST**	**2.24%**	**6.86%**	**0**
**KMT2C**	**3.04%**	**6.57%**	**4.3%**
**LRP2**	**2.46%**	**6.42%**	**2.8%**
**MACF1**	**1.73%**	**5.84%**	**0**
**TP53**	**8.31%**	**5.26%**	**7.2%**
**PKHD1**	**1.82%**	**5.26%**	**0**
**MTOR**	**4.67%**	**5.11%**	**4.8%**
**CUBN**	**1.96%**	**5.11%**	**0**
**OBSCN**	**1.51%**	**4.96%**	**0**
**PTEN**	**3.16%**	**4.67%**	**3.8%**
**USH2A**	**2.01%**	**4.67%**	**0**
**PCLO**	**2.83%**	**4.67%**	**3.7%**
**KMT2D**	**2.18%**	**4.67%**	**3.7%**
**SYNE1**	**2.33%**	**4.53%**	**0**
**FAT1**	**2.07%**	**4.53%**	**3.1%**
**CSMD3**	**2.67%**	**4.38%**	**0**
**ARID1A**	**2.57%**	**4.38%**	**3.2%**
**UBR4**	**1.83%**	**4.38%**	**0**
**HMCN1**	**1.92%**	**4.38%**	**0**

### PARPi Suppress Cell Survival of RCC Cells

**Figure 1:**
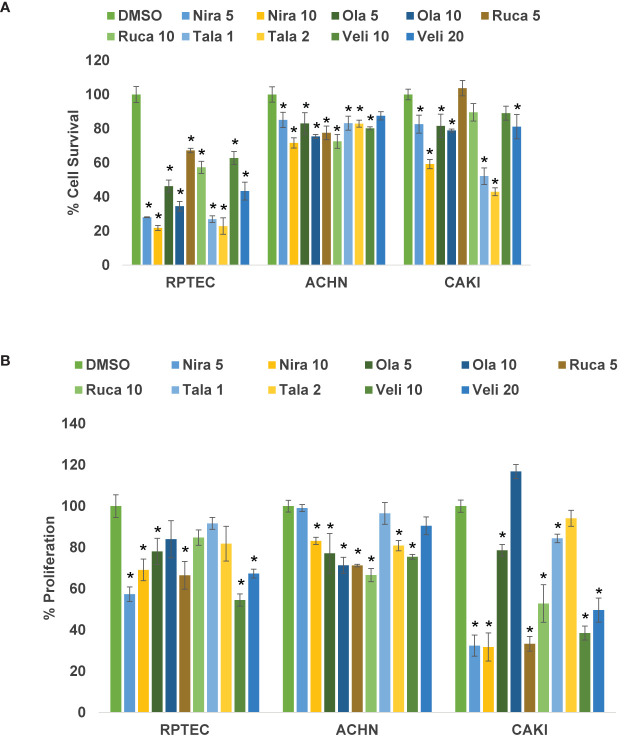
**(A)** PARPi suppressed the survival of human RCC cells. RPTEC/TERT1, ACHN, and Caki-2 were treated with 5 or 10 μM each of niraparib, olaparib, or rucaparib; 1 or 2 μM talazoparib; or 10 or 20 μM veliparib for 72 h and cell survival was measured by cell counting (Beckman Coulter). Cell survival was calculated as the number of cells remaining attached in each treatment compared with the control (DMSO). Results are presented as means ± SD of 3 separate experiments with triplicates. P < 0.05 was considered significant (*). Niraparib and talazoparib were effective in reducing the cell survival of RCC cells. **(B)** PARPi inhibited the proliferation of RCC cells. RPTEC/TERT1, ACHN, and Caki-2 cells were treated with 5 or 10 μM each of niraparib, olaparib, or rucaparib; 1 or 2 μM talazoparib; or 10 or 20 μM veliparib for 72 h and cell proliferation was measured using the CellTiter 96 cell proliferation assay (Promega). Cell proliferation is reported as % cell proliferation compared with control (DMSO). Results are presented as means ± SD of 3 separate experiments with triplicates. P< 0.05 was considered significant (*). Niraparib, rucaparib, and talazoparib were effective in reducing the proliferation of RCC cells.

We treated the RCC cell lines Caki-2 and ACHN as well as the normal renal epithelial cell line RPTEC/TERT1 with varying concentrations of PARPi (niraparib, olaparib, rucaparib, talazoparib, and veliparib) for 0-72 h. As shown in **Figure 1A**, niraparib, olaparib, rucaparib, and talazoparib suppressed the cell survival of Caki-2 and ACHN cells significantly compared to cells treated with DMSO. Veliparib was not effective in achieving >30% inhibition of cell growth even when used at very high concentrations (100μM). According to our results, niraparib and talazoparib were the most effective in achieving >50% suppression of cell survival with 72 h treatment. Caki-2 cells were found to be more sensitive to PARPi compared with ACHN cells, indicating that ccRCC may be more amenable to treatment with PARPi.

### PARPi Suppress Cell Proliferation of RCC Cells

We treated RCC cell lines Caki-2 and ACHN and the normal renal epithelial cells with varying concentrations of PARPi for 2-5 days. As shown in **Figure 1B**, niraparib, rucaparib, and talazoparib suppressed the proliferation of RCC cells up to 70%, compared with that of RPTEC/TERT1 cells. These results indicated that PARPi can be used as potential therapeutic agents against RCC. As noted above, proliferation of Caki-2 cells was preferentially suppressed by PARP inhibition, compared with that of ACHN cells, confirming that PARPi may be effective therapeutic agents for ccRCC.

### PARPi Suppress the Clonogenic Ability of RCC Cells

**Figure 2:**
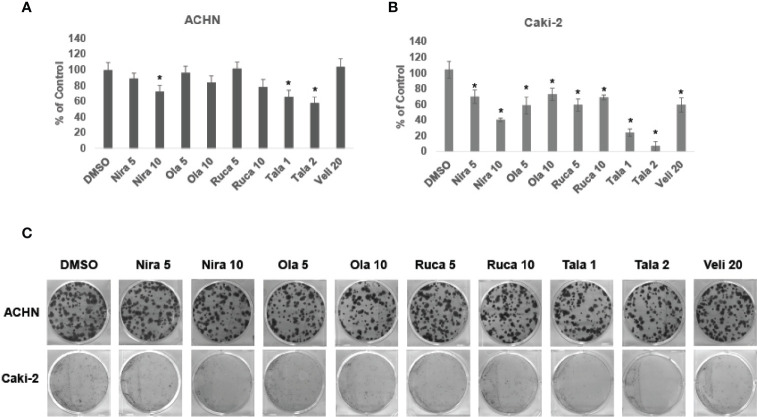
PARPi inhibited the clonogenic ability of RCC cells. **(A)** ACHN and **(B)** Caki-2 cells were treated with 5 or 10 μM each of niraparib, olaparib, or rucaparib; 1 or 2 μM talazoparib; or 20 μM veliparib for 72 h. Cell were trypsinized and plated at low density (400 cells/well) in 6-well plates. Plates were left undisturbed for 10-14 days and the resulting colonies were stained with 0.5% crystal violet in buffered formalin. Colonies were counted using the ImageJ Colony Counter plug-in. Results are presented as means ± SD of 3 separate experiments with triplicates. P< 0.05 was considered significant (*). **(C)** Representative images of colonies formed by ACHN and Caki-2 cells in the different treatments are shown. Niraparib, Olaparib, and talazoparib were effective at reducing the clonogenic ability of ACHN and Caki-2 cells. Caki-2 cells were more sensitive to treatment with PARPi.

We treated RCC cell lines ACHN and Caki-2 with different concentrations of PARPi for 72 h and performed clonogenic assays as described earlier. The results showed that PARPi inhibited the clonogenic ability of ACHN (**Figures 2A, C**) and Caki-2 (**Figures 2B, C**) cells significantly. RPTEC/TERT1 cells failed to form colonies in this assay (results not shown). These results collectively indicated that PARPi can prevent colony formation by RCC cells.

### PARPi Induce Apoptosis in RCC Cells

Cell lysates from RPTEC/TERT1, Caki-2, and ACHN cells treated with varying concentrations of niraparib, olaparib, rucaparib, talazoparib, or veliparib were analyzed using Western blotting to assess the levels of apoptosis. Levels of cleaved caspases 3, 7, and 9, as well as levels of cleaved PARP were analyzed. The results showed that treatment with PARPi increased the levels of cleaved caspase 9 and cleaved PARP in Caki-2 cells more significantly compared with those in ACHN cells (**Supplementary Figure 1**). These findings indicate that PARP inhibition was successful in inducing apoptosis in ccRCC cells.

### PARPi Suppress RCC Xenograft Growth *In Vivo*

**Figure 3:**
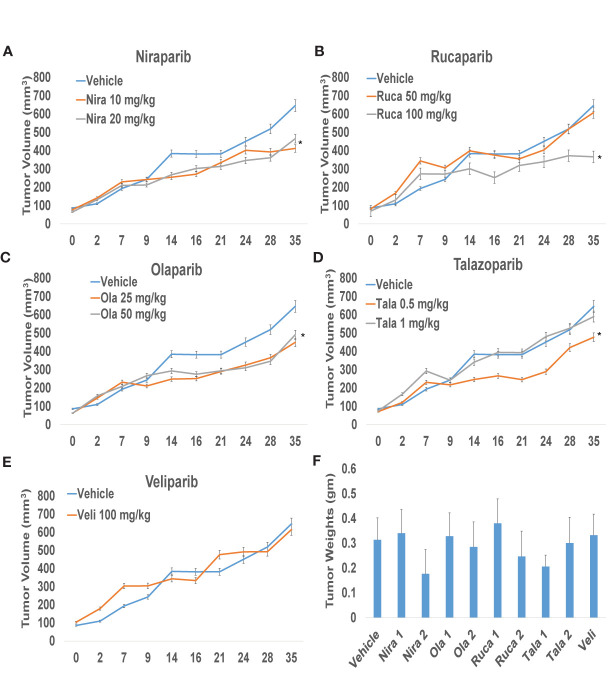
PARPi suppressed RCC xenograft growth. RCC xenografts in both flanks of male SCID mice (mouse n = 5; tumor n = 10) were treated *via* oral gavage with vehicle (0.5% Methocel A4M), or the indicated doses of PARPi: 10 or 20 mg/kg niraparib **(A)**; 25 or 50 mg/kg olaparib **(B)**; 50 or 100 mg/kg rucaparib **(C)**; 0.5 or 1 mg/kg talazoparib **(D)**; or 100 mg/kg veliparib **(E)**. Tumor volumes were measured twice weekly with digital calipers. Values are presented as average tumor volumes ± SD. P < 0.05 was considered significant (*). Tumor weights at the end of the experiment were measured **(F)**. Both doses of niraparib and olaparib along with one dose each of rucaparib and talazoparib were effective in reducing the growth rate of RCC xenografts. Veliparib was not able to reduce RCC tumor xenograft growth.

**Figure 4:**
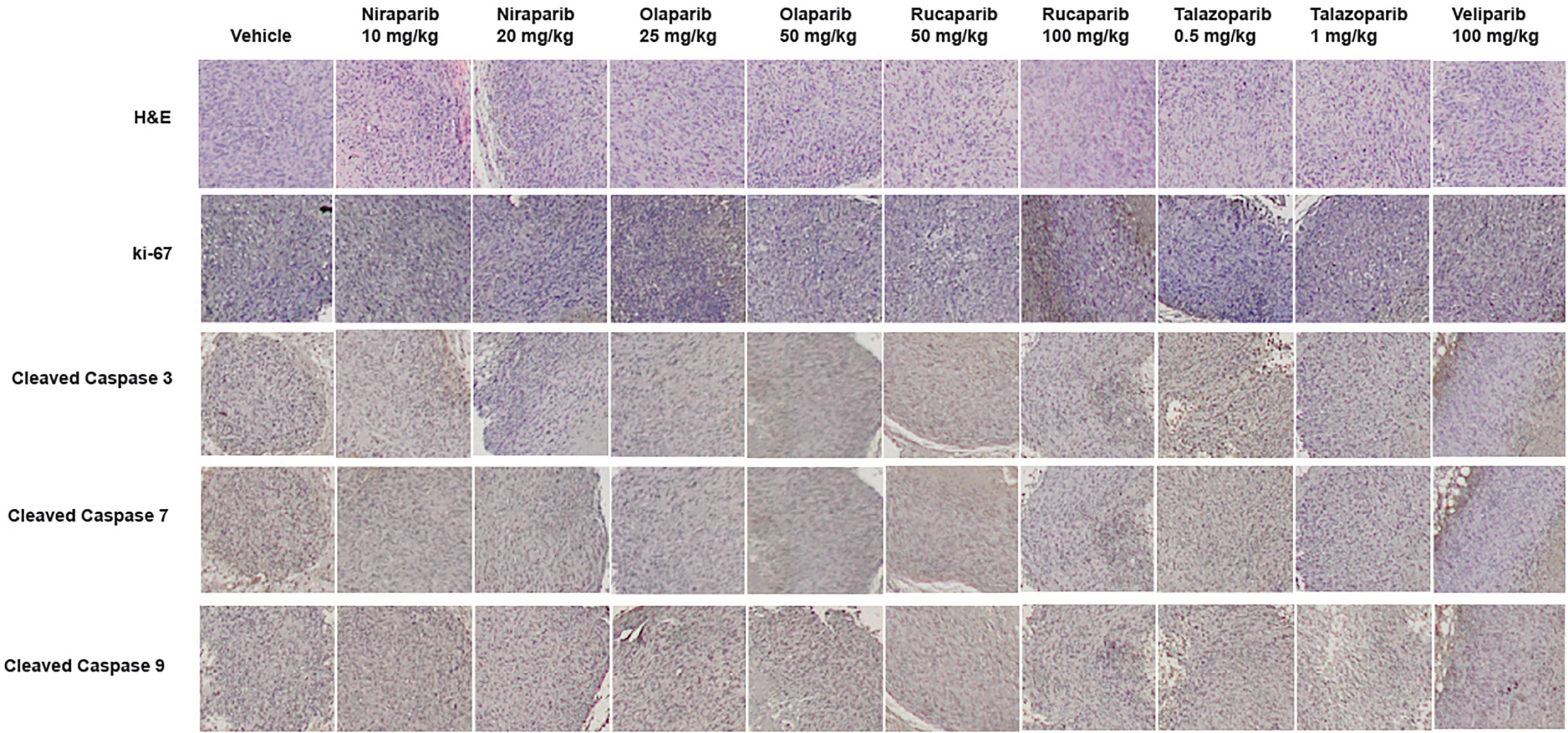
Treatment with PARPi induced apoptotic markers in RCC xenografts. RCC xenograft tissues were subjected to immunohistochemical analyses using antibodies against the proliferation marker ki-67 and the apoptotic markers cleaved caspases 3, 7, and 9. Representative images are shown in each group. Treatment with PARPi inhibited ki-67 and induced higher levels of cleaved caspases 3, 7, and 9.

To confirm that PARPi can reduce the growth of RCC xenografts *in vivo*, we generated RCC xenografts in SCID mice and treated them with PARPi (niraparib, olaparib, rucaparib, talazoparib, or veliparib) for ~5 weeks. We monitored the mice for weight loss and examined sera for ALP or AST activity to assess the toxicity effects of PARPi. At the end of the experiment, mice were euthanized, and tumor tissues were collected. Formalin-fixed paraffin-embedded sections of the tumor tissues were analyzed by immunohistochemistry for the expression of ki-67, a proliferation marker, and cleaved caspases 3, 7, and 9 to assess tumor cell proliferation and apoptosis upon treatment with the various compounds. The results (**Figures 3A–D**) illustrated that niraparib (at both doses used 10 mg/kg and 20 mg/kg), rucaparib (at the higher dose of 100 mg/kg), olaparib (at both doses used 25 mg/kg and 50 mg/kg), and talazoparib (at the lower dose of 0.5 mg/kg) were successful in reducing the growth rate of RCC xenografts. Veliparib, as predicted based on *in vitro* results, was not able to reduce the growth rate of RCC xenografts (**Figure 3E**). These results were reflected in the tumor weights measured at the end of the experiment (**Figure 3F**). No appreciable toxicity effects such as weight loss were observed in the mice treated with PARPi. Immunohistochemical analyses revealed that the proliferation marker ki-67 was downregulated in xenografts treated with PARPi, while levels of cleaved caspases 3, 7, and 9 were elevated (**Figure 4**), indicating that PARPi induced apoptosis in the treated xenografts.

Taken together, our results demonstrated that PARPi induce apoptosis, reduce cell growth and proliferation in RCC cells, and may be used as effective therapeutic agents against RCC.

## Discussion

The principal strategy for the management of non-metastatic RCC (nmRCC) is definitive local treatment. However, up to 40% of patients will develop mRCC even after treatment for localized disease (22, 23). Until recently, vascular endothelial growth factor receptor (VEGF) targeting therapies, such as sunitinib or pazopanib, were considered first-line standard of care for mRCC (24). Inhibitors of the mTOR pathway have also been used in the management of mRCC. However, despite initial clinically significant response rates and improved outcomes, resistance to tyrosine kinase inhibitors (TKIs) and mTOR inhibitors develops in nearly all patients (25). A new paradigm in the treatment of mRCC has emerged in recent years with the establishment of the role of the immune system in ccRCC biology. Hence, immune checkpoint inhibitors (CPIs), such as nivolumab, pembrolizumab, and ipilimumab, now constitute the mainstay of mRCC treatment (26, 27). Several immunotherapy combinations such as nivolumab plus ipilimumab; pembrolizumab plus axitinib; avelumab plus axitinib; or atezolizumab plus bevacizumab have proved successful in overcoming drug resistance and achieving a higher objective response rate (28), and have since become the current standard of care for mRCC. Despite these advances, the potential of resistance and the lack of biomarkers to predict responses or outcomes remains a major challenge. Consequently, novel drugs with new targets and mechanisms of action are needed.

Prior studies have found high rates of alterations in DNA damage repair genes in localized and metastatic RCC tissues (18, 19). In the current study, we used results from literature as well as our data-mining results showing that RCC tissues harbor mutations in several DNA damage repair (HR) genes as rationale to test whether PARP inhibition is a rational therapeutic strategy against RCC. A few previous studies have examined the efficacy of PARP inhibition in VHL-deficient RCC cells (29) and in hereditary cancer syndromes (30) using olaparib and BMN-673 (talazoparib). In this study, we compared the relative efficacy of 5 commercially available PARPi in ccRCC and pRCC cell lines.

PARPi mechanism of action may include two mechanisms: catalytic inhibition of PARP or “PARP trapping” (PARP is trapped at sites of DNA damage), leading to prevention of repair and cytotoxicity (31). Upon treatment of ccRCC and pRCC cells with 5 different commercially available PARPi, we found that ccRCC may be highly sensitive to PARP inhibition. Specifically, our results demonstrated that PARPi with higher PARP trapping activity such as niraparib, olaparib, and talazoparib are more effective at reducing the cell survival, proliferation, clonogenic ability, and xenograft growth of RCC cells compared to those (veliparib) that preferentially inhibit PARP catalytic activity. These findings may have significant implications in choosing potential agents for RCC therapy in the future. Further, PARP inhibition may confer synthetic lethality in tissues that harbor mutations in DNA damage repair genes such as BRCA or other HR genes.

The PARPi olaparib and talazoparib have been approved as single agents in treating metastatic breast cancers that have a BRCA mutation (32). Olaparib, rucaparib, and niraparib have been approved for advanced ovarian, primary peritoneal or Fallopian tube cancers with BRCA mutations (33). Olaparib has also been approved for the treatment of pancreatic cancers with BRCA mutations (34). Very recently, Olaparib and rucaparib have been approved for the treatment of metastatic castration resistant prostate cancer (35). Several clinical trials are examining PARPi in RCC with DNA damage repair gene mutations (NCT03786796; NCT04337970).

In summary, we present evidence that PARP inhibition with niraparib, rucaparib, and talazoparib demonstrated superior *in vitro* and *in vivo* antitumor activity in RCC cells and xenografts. To our knowledge, ours is one of the first studies to compare the effects of all commercially available PARPi against RCC cells. Our findings suggest that PARPi may represent a rational therapeutic strategy to treat RCC and may in future be considered as a part of combinatorial approaches to overcome resistance to established therapeutics in mRCC. Our current study examined the potential efficacy of PARPi as single agents against RCC, but studies are ongoing to unravel the utility of PARPi in combination with TKI or immunotherapy.

## Data Availability Statement

The datasets presented in this study can be found in online repositories. The names of the repository/repositories can be found in the article.

## Ethics Statement

The animal study was reviewed and approved by the Institutional Animal Care and Use Committee, University of Toledo Health Science Campus.

## Author Contributions

Conceptualization: NN and FP. Methodology: NN and JP. Data acquisition: JP, SB, JD, and RW. Validation: JP and SB. Resources: NN, PS, and FP. Writing – Original Draft Preparation: NN and FP. Writing – Review & Editing, JP, JD, and PS. Supervision: NN. Project Administration: NN and FP. Funding Acquisition: NN and FP. All authors contributed to the article and approved the submitted version.

## Funding

This work was supported in part by NCI grants R21CA203406, R03CA198696, and the College of Medicine and Life Sciences, University of Toledo (NN) and an Endowed Chair from the Stranahan Foundation for Oncological Research, Toledo, Ohio (FP).

## Conflict of Interest

The authors declare that the research was conducted in the absence of any commercial or financial relationships that could be construed as a potential conflict of interest.

## Supplementary Material

Supplementary Figure 1 PARPi induced apoptosis in RCC cells. RPTEC/TERT1, ACHN, and Caki-2 cells were treated with 5 or 10 μM each of niraparib, olaparib, or rucaparib; 1 or 2 μM talazoparib; or 10 or 20 μM veliparib for 72 h and the cell lysates were subjected to Western blotting with antibodies against cleaved caspases 3, 7, 9; cleaved PARP; whole caspases 3, 7, 9; and whole PARP. Tubulin was used as the loading control. Results are representative images from 3 separate experiments with duplicates. Niraparib, olaparib, rucaparib, and talazoparib induced apoptosis in RCC cells.
