# Supplementary figures and images for: The Emerging Role of Poly (ADP-Ribose) Polymerase Inhibitors as Effective Therapeutic Agents in Renal Cell Carcinoma
Source: Front Oncol. 2021 Jul 9;11:681441. doi: 10.3389/fonc.2021.681441 (PMC8300201; doi:10.3389/fonc.2021.681441)

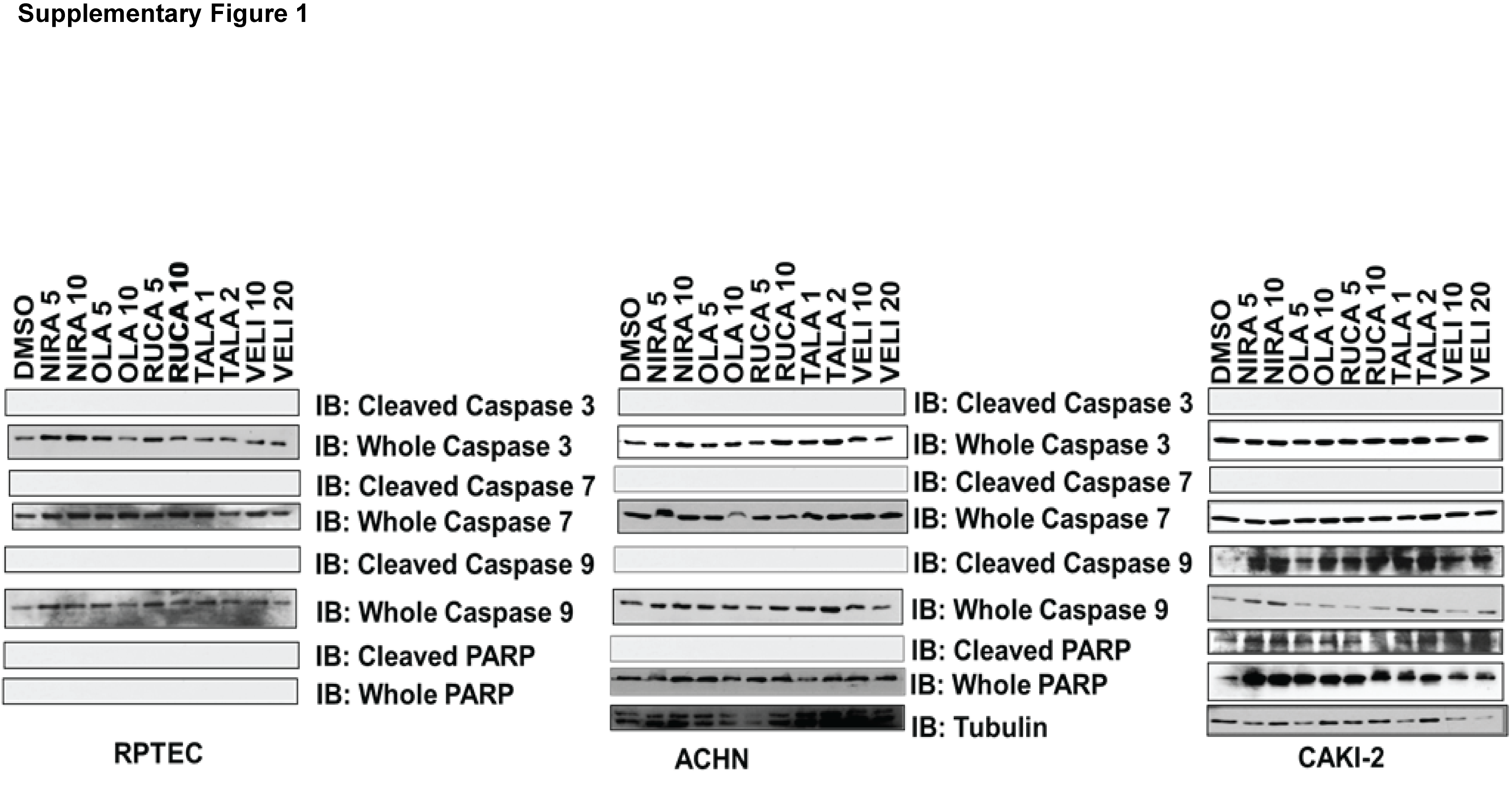

Supplement: Supplementary Figure 1 — PARPi induced apoptosis in RCC cells. RPTEC/TERT1, ACHN, and Caki-2 cells were treated with 5 or 10 μM each of niraparib, olaparib, or rucaparib; 1 or 2 μM talazoparib; or 10 or 20 μM veliparib for 72 h and the cell lysates were subjected to Western blotting with antibodies against cleaved caspases 3, 7, 9; cleaved PARP; whole caspases 3, 7, 9; and whole PARP. Tubulin was used as the loading control. Results are representative images from 3 separate experiments with duplicates. Niraparib, olaparib, rucaparib, and talazoparib induced apoptosis in RCC cells. [file Image_1.tif]
